# Health of the World

**Published:** 1924-03

**Authors:** 


### HEALTH OF THE WORLD.

The Health Committee of the League of Nations began its new session in Geneva on February 11. This meeting, from the point of view of work, is a direct continuation of the six sessions already held by the Provisional Health Committee of the League. From the constitutional point of view, however, it is the first meeting of the Health Committee of the permanent Health Organisation that was formed at the end of last year, as a result of an agreement concluded with the Office International d'Hygiène Publique at Paris and the Provisional Health Committee of the League.

### Constitution of the Committee.

In this new organisation the Comité Permanent of the Office International d'Hygiène Publique, which retains its functions and constitution intact, acts as the Advisory Council of the League Health Organisation, and elects nine members of the League Health Committee. The President of the Office International d'Hygiène Publique is also ex-officio a member of the Health Committee. The Council of the League appoints a further six members of the Health Committee. The standing Health Committee thus constituted may be supplemented by the addition of not more than four public health experts as assessors, who will be appointed by the League Council on the nomination of the Health Committee, and will be considered as fully effective members. The Health Section of the Secretariat of the League forms as hitherto the Secretariat of the League Health Organisation.

### Opium Traffic and Cancer Statistics.

Several sub-committees are reporting on the work that has been done since the last meeting of the Health Committee in June, 1923. Thus the Opium Sub-Committee—made up of members of the Opium and Health Committees—has undertaken an inquiry into the average amounts of opium and dangerous drugs required by each country annually for medical and scientific purposes, and the best way of pursuing further inquiries into this subject. Their conclusions will now be examined by the full Health and Opium Committees, respectively, and in the form in which they are approved will be used by the Opium Committee in the course of its work on the control of traffic in opium and dangerous drugs. The Cancer Sub-Committee of the Health Committee will submit a plan of inquiry into the vital statistics of certain countries, for the purpose of discovering the reasons for the wide variance in cancer mortality.

### Incidences of Malaria.

The Malaria Sub-Committee will also submit a report. This sub-committee was set up at the last meeting of the Health Committee in order to examine the information collected with regard to the incidence of malaria throughout the world, particularly in Europe, and the different prophylactic measures used, or which might be used, to combat this disease, as well as to prepare replies to questions on these problems which might be asked by the Health Administrations of the different States. This inquiry was undertaken owing to the menacing spread of malaria in Eastern and Southern Europe since the war. Thus the Albanian Government has requested technical aid in preparing an anti-malaria campaign in that country. A member of the Epidemic Commission, Dr. Haigh, who was sent to Albania to investigate the situation, has made a preliminary inquiry into the prevalence of malaria in that country, which will be submitted to the committee. A request by the Greek Government for technical advice in organising an anti-malaria campaign in Greece will also be considered.

### The Interchange of Medical Officers.

Further plans for "interchanges" of medical health personnel during the coming year will be considered. At present a collective interchange is taking place in Great Britain, a second has been planned for the Netherlands and Denmark, a third for Switzerland, and a fourth may be organised particularly for the countries of the Far East. Every effort is also being made to profit by past experience in order to perfect in detail the manner in which these interchanges are conducted so as to make them of the greatest value to all concerned.

---

**Cinematograph for a Mental Hospital.**

Mr. Thomas Field, chairman of the visiting committee of the Bucks County Mental Hospital, and his sister have presented the institution with a cinematograph, a portable wireless installation and a Marconi amplifier for the amusement of the patients. Mr. Field is the founder of the Mental Hospitals' Association.

